# Effectiveness and Safety of Dupilumab in Patients with Chronic Rhinosinusitis with Nasal Polyps and Associated T2 Comorbidities: One-Year Real Life Round

**DOI:** 10.3390/jcm15041373

**Published:** 2026-02-10

**Authors:** Eustachio Nettis, Rossella Casella, Elisabetta Di Leo, Ippolita Zaza, Fabio Lodi Rizzini, Alessandro Vrenna, Luisa Brussino, Irene Ridolfi, Laura Bonzano, Lia Ginaldi, Ernesto Aitella, Vincenzo Patella, Roberta Zunno, Massimo Triggiani, Isabella Carrieri, Nicola Antonio Adolfo Quaranta, Lucia Iannuzzi, Francesca Serena Romano, Erminia Ridolo, Alessandro Barone, Angela Maria D’Uggento, Valentina D’Aiuto, Aikaterini Detoraki

**Affiliations:** 1 Department of Emergency and Organ Transplantation, School of Allergology and Clinical Immunology, University of Bari Aldo Moro, Policlinico di Bari, 70124 Bari, Italy; 2 Section of Allergy and Clinical Immunology, Unit of Internal Medicine, “F. Miulli” Hospital, Strada Provinciale per Santeramo Km 4.100, 70021 Acquaviva delle Fonti, Italy; 3 Facoltà Medicina e Chirurgia, Università Studi Brescia, SSVD Allergologia—Spedali Civili Brescia, 25123 Brescia, Italy; 4 Allergy and Clinical Immunology Unit, Department of Medical Sciences, University of Torino & Mauriziano Hospital, 10128 Turin, Italy; 5 Dermatology Unit, Azienda Unità Sanitaria Locale-Istituto di Ricovero e Cura a Carattere Scientifico di Reggio Emilia, 42122 Reggio Emilia, Italy; 6 Department of Life, Health and Environmental Sciences, University of L’Aquila, 67100 L’Aquila, Italy; 7 Allergy and Clinical Immunology Unit, Center for the Diagnosis and Treatment of Osteoporosis, Azienda Unità Sanitaria Locale 04 Teramo, 64100 Teramo, Italy; 8 Division Allergy and Clinical Immunology, Department of Medicine Azienda Sanitaria Locale Salerno, “Santa Maria della Speranza” Hospital, 84131 Salerno, Italy; 9 Department of Medicine, Surgery and Dentistry, University of Salerno, 84084 Salerno, Italy; 10 Otolaryngology Unit, Department of Basic Medical Science, Neuroscience and Sensory Organs, University of Bari Aldo Moro, 70124 Bari, Italy; 11 Department of Medicine and Surgery, University of Parma, 43126 Parma, Italy; 12 Department of Economics and Finance, University of Bari “Aldo Moro”, 70124 Bari, Italy; 13 Department of Internal Medicine and Clinical Complexity, Azienda Ospedaliera Universitaria Federico II, 80131 Napoli, Italy

**Keywords:** chronic rhinosinusitis with nasal polyps, dupilumab, type 2 inflammation, atopic comorbidities, multimorbidity, real-world study, asthma, atopic dermatitis, biologic therapy, precision medicine

## Abstract

**Background/Objectives:** Chronic rhinosinusitis with nasal polyps (CRSwNP) represents a common and debilitating inflammatory disorder primarily driven by type 2 immune mechanisms. Its frequent overlap with asthma, allergic rhinoconjunctivitis and atopic dermatitis highlights the need for therapeutic strategies able to address multimorbidity within the same pathogenic spectrum. The development of monoclonal antibodies targeting signaling pathways provides an effective and well-tolerated option that addresses common comorbidities. Targeting the IL-4 receptor alpha subunit, dupilumab is a completely human IgG4 monoclonal antibody that reduces type 2 inflammation in many organ systems by blocking IL-4 and IL-13 signaling. This study aimed to assess the long-term effectiveness and safety of dupilumab in a real-world cohort of patients with severe CRSwNP, stratified according to the presence of common type 2 comorbidities, over a 52-week treatment period. **Methods:** We conducted a prospective, multicenter, observational study across ten Italian secondary care centers for Allergy and Clinical Immunology and Otolaryngology. All participating centers were affiliated with the Italian Society of Allergy, Asthma and Clinical Immunology (SIAAIC). Enrolled adult subjects with severe CRSwNP received dupilumab treatment in the context of standard care for 52 weeks. Several efficacy parameters were used. **Results:** A significant improvement was detected for all the applied efficacy parameters, i.e., 22-item Sinonasal Outcome Test (SNOT-22) and bilateral endoscopic nasal polyp (NPS) scores for CRSwNP, Rhinitis Control Scoring System (RCSS) and Rhinoconjunctivitis Quality of Life Questionnaire (RQLQ) scores for allergic perennial rhinitis, Forced Expiratory Volume in the first second (FEV1) and Asthma Quality of Life Questionnaire (AQLQ) scores for asthma, Eczema Area and Severity Index (EASI), Atopic Dermatitis Control Toll (ADCT) and Dermatology Life Quality Index (DLQI) scores for AD. Dupilumab was well-tolerated, with no new safety signals. **Conclusions:** This multicenter real-world study demonstrates that dupilumab provides sustained, clinically meaningful, and safe benefits for patients with severe CRSwNP and coexisting type 2 comorbidities, supporting its role as an integrated therapeutic option in precision management of type 2 inflammatory diseases.

## 1. Introduction

Chronic rhinosinusitis with nasal polyposis (CRSwNP) is a major healthcare challenge, affecting 5 to 28% of the worldwide population. The disease is recognized as a mani-festation primarily driven by type 2 inflammation [1]. Epidemiological and clinical studies consistently report a high prevalence of overlapping atopic comorbidities in patients with CRSwNP, reflecting the shared immunopathogenic mechanism underlaying these conditions [2]. Bronchial asthma represents the most frequent association, affecting approximately 40–60% of patients with CRSwNP [3], while allergic rhinitis is reported in up to 50–80% of cases [4]. In addition, atopic dermatitis has been described in around 10–25% of patients [5]. Conversely, data on the association between CRSwNP and chronic spontaneous urticaria are scarce and inconsistent [6].

The frequent coexistence of upper and lower airway disease and skin involvement highlights the burden of atopic multimorbidity and the need for therapeutic strategies capable of targeting shared pathogenic pathways and offering collateral benefits [5]. In this context, the development of monoclonal antibodies, targeting signaling pathways, represents an effective and safe treatment approach for patients with CRSwNP and atopic comorbidities.

Dupilumab, a fully human monoclonal antibody directed against the IL-4 receptor alpha chain, blocks the activity of both IL-4 and IL-13, two key cytokines of type 2 immune responses [7].

We previously demonstrated the rapid effectiveness of dupilumab in real-life conditions in patients with perennial allergic rhinitis (PAR) and/or asthma and/or AD and/or CSU associated with CRSwNP for 16 weeks [8].

The aim of this study was to describe the effectiveness and safety of dupilumab in a real-world setting in the management of patients with CRSwNP stratified by common overlapping T2 comorbidities during 52 weeks of treatment.

## 2. Materials and Methods

### 2.1. Study Design and Population

We performed a 52-week, multicenter, prospective, observational study recruiting adult patients (≥18 years of age) with severe CRSwNP under treatment with dupilumab as part of standard care.

The data were collected from 10 Italian care centers for Allergy and Clinical Immunology and Otolaryngology, all of which were members of SIAAIC (Italian Society of Allergy, Asthma and Clinical Immunology).

Inclusion criteria for this study were: age ≥18 years; diagnosis of severe chronic rhinosinusitis with nasal polyps (CRSwNP), confirmed by Nasal Polyps Score (NPS) ≥ 5 and/or Sinonasal Outcome Test (SNOT-22) ≥ 50; evidence of type 2 inflammation, defined by blood eosinophil counts > 150 cells/μL or tissue eosinophils ≥ 10 per high-power field (HPF) and/or total IgE ≥ 100; persistent symptoms despite treatment with intranasal corticosteroids (INS); failure of, or contraindication, or intolerance to previous medical treatments, including at least two cycles of systemic corticosteroid over the last year, and/or history of endoscopic sinus surgery (ESS), in accordance with the Italian Agency of Drugs (AIFA) guidelines and the EPOS/EUFOREA update [2,9,10]. Exclusion criteria were: age < 18 years; pregnancy and breastfeeding; and ESS in the prior 3 months.

At baseline, patients who met the inclusion criteria were assessed for medical history, demographics, comorbid diseases (i.e., allergic rhinoconjunctivitis, bronchial asthma, atopic dermatitis, chronic spontaneous urticaria), and concomitant medications or procedures. A complete physical examination was performed.

All patients received a loading dose of dupilumab 300 mg subcutaneously administered by a clinician, followed by dupilumab 300 mg every other week for 52 weeks. Throughout the study period, patients would maintain their pre-treatment medication for the management of CRSwNP and other comorbidities. Given the results of our previous observational 16 weeks real-life study showing rapid effectiveness of dupilumab in patients with perennial allergic rhinitis and/or asthma and/or AD and/or CSU associated with CRSwNP [8], in this study we chose to explore a longer period of treatment; therefore, effectiveness was evaluated after 52 weeks. Accordingly, drug safety was assessed by recording and monitoring adverse events.

### 2.2. Ethical Considerations

The study was conducted following the Declaration of Helsinki and was approved by the Institutional Review Board of “Federico II” University Hospital (Prot.75/21, data of approval: 6 May 2021). Informed consent was obtained from all patients who agreed to participate to this study.

### 2.3. Procedures, Outcomes and Statistical Analysis

To evaluate CRSwNP patients, outcome measures at baseline and after 52 weeks included:

- Nasal Polyps Score (NPS), endoscopic assessment of polyp size (0–4 per nostril; total score 0–8) [11].
- 22-item Sinonasal Outcome Test (SNOT-22), validated questionnaire measuring sinonasal symptoms and quality of life (0–110) [12].
- Nasal congestion score, which assess congestion and obstruction recalled over the past 24 h, using a 0-to-3-point scale [13].
- Loss of smell score (LOS), which assessed the loss of smell using a patient-reported daily diary with a scale of 0 to 3, where 0 = no symptom, 1 = mild LoS, 2 = moderate LoS, and 3 = severe LoS [14].
- Visual analog scale (VAS): global assessment of disease severity and smell impairment (0–10) [13].
- Rhinitis Control Scoring System (RCSS), validated 10-item questionnaire (score range 10–50) assessing rhinitis control, and Rhinoconjunctivitis Quality of Life Questionnaire (RQLQ), which evaluate disease-specific quality of life through a 28-item questionnaire (0–6) [8].
- Asthma outcomes: Spirometry (FEV1 and % predicted), Asthma Control Test (ACT, 0–25), and standardized Asthma Quality of Life Questionnaire (AQLQ[S], 0–7) [15,16].
- Atopic dermatitis outcomes: Eczema Area and Severity Index (EASI, 0–72), Atopic Dermatitis Control Tool (ADCT, 0–24), pruritus and sleep disturbance assessed with Numerical Rating Scales (NRS, 0–10), and Dermatology Life Quality Index (DLQI, 0–28) [17,18,19].
- Chronic spontaneous urticaria: Urticaria Activity Score over 7 days (UAS7, 0–42) [20].

Additional evaluations included skin prick testing to inhalant and food allergens, serum total IgE (normal <100 kU/L), and peripheral blood eosinophil counts (normal <500 cells/mm^3^).

To compare the data collected for the same patient at the two time points, the non-parametric Wilcoxon test for related samples was used, as the data were not normally distributed. The main descriptive data (mean, standard deviation, median) were also calculated for all patients. Regression analysis with backward criterion was performed to understand which clinical variables had the greatest impact on patient health improvement. The statistical analyses were performed using IBM SPSS Version 26 software (IBM, Armonk, NY, USA, 2017) [21].

## 3. Results

In total, 122 patients with nasal polyposis were identified and received treatment with dupilumab. Baseline demographics and characteristics are summarized in Table 1.

The study cohort included 58 males (51%) with a median age ± interquartile range (IQR) of 50 ± 17 years. The median ± IQR body mass index was 25 ± 5.0 kg/m^2^. The median duration of nasal polyps was 84 months (IQR 132), and the majority of patients (84.1%) had undergone at least one prior surgical intervention, while 17.7% had received three or more surgeries. The median ± IQR endoscopic NPS was 5.5 ± 2.25 while SNOT-22 score was 63 ± 34. A total of 39 patients (34.5%) had positive prick test results. Allergic rhinoconjunctivitis was documented in 52.4% of the cohort, while 9.8% presented with non-allergic rhinoconjunctivitis. Bronchial asthma was the most frequent associated condition, affecting 54.1% of patients, followed by atopic dermatitis in 15.6% and food allergy in 4.1% of cases. No patients were diagnosed with chronic spontaneous urticaria.

Marked clinical improvement was observed in all sinonasal parameters after 52 weeks of treatment. The NPS and SNOT-22 score significantly decreased from baseline to week 52 (median ± IQR = 1 ± 2 and median ±  IQR = 13 ± 13, respectively, *p* < 0.001) (Table 2). A total of 90 patients (73.77%) achieved a score between 0 and 1 in NPS at week 52. All patients (100%) had a clinically meaningful improvement in SNOT-22 score (≥8.9 points).

**Figure 1:**
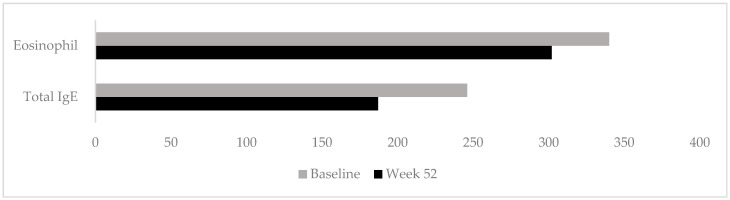
Comparison of the median values of total IgE and eosinophil outcomes measured at baseline and at week 52.

The value of NC score (median ± IQR: 1 ± 1), of LoS score (median ± IQR: 1 ± 1) and of anterior/posterior rhinorrhea score (median ± IQR: 0 ± 1), patient-reported total symptom score (median ± IQR: 1 ± 2), and smell capacity assessed with VAS (median ± IQR: 1 ± 3) each showed a significant decrease from baseline to week 52 (*p* < 0.001). Median serum total IgE levels significantly decreased from 246 to 187 kU/L (*p* = 0.003) at week 52. Conversely, peripheral blood eosinophil counts did not differ significantly from baseline (median 340 vs. 302 cells/mm^3^; *p* = 0.951), as shown in Table 2 and Figure 1.

A backward stepwise multiple linear regression analysis was performed to identify the clinical variables most strongly associated with changes in disease burden after 52 weeks of treatment. The dependent variable was defined as the change in SNOT-22 score (ΔSNOT-22), calculated as the difference between week 52 (T52) and baseline (T0) values. Independent variables (regressors) included the corresponding changes (Δ) in key clinical parameters—total endoscopic nasal polyp score (TENPS), patient-reported total symptom score (POEMS), total serum IgE levels, nasal congestion score (NCS), blood eosinophil count, and visual analog scale (VAS) for disease severity—as well as demographic and clinical covariates (age, sex, disease duration, and number of previous surgeries). In the initial model, all variables were included; non-significant predictors were sequentially removed according to the backward elimination procedure. The final model demonstrated a good overall fit (R^2^ = 0.65) and identified four variables that were independently associated with ΔSNOT-22 (*p* ≤ 0.01) (Table 3). Interpretation of the model suggests that improvement in patient-reported total symptom burden (ΔPOEMS) and reduction in endoscopic polyp size (ΔTENPS) were the strongest independent predictors of SNOT-22 improvement. Notably, an inverse relationship was observed between ΔVAS and ΔSNOT-22, indicating that reductions in patient-perceived disease severity correlated with greater improvements in sinonasal-specific quality of life. Although the reduction in peripheral blood eosinophil count (Δeosinophils) also reached statistical significance, its standardized beta coefficient was relatively low, suggesting a more modest contribution to the overall variance explained by the model. These findings support the concept that both objective endoscopic improvements and subjective symptom relief are critical determinants of patient-perceived disease control.

### 3.1. T2 Comorbidities

Among 76 patients with rhinoconjunctivitis, 64 (52.4%) had perennial allergic rhinitis confirmed by history and skin testing. Significant improvements were observed in RCSS (median 22.5 → 12.0, *p*< 0.001) and RQLQ (median 3.0 → 0.9, *p* < 0.001) (Table 4).

Fifty-eight patients (47.5%) were diagnosed with asthma according to GINA criteria (Table 4) [22]. All patients in this subgroup had received asthma medications, primarily inhaled corticosteroids and long-acting β-agonists in the previous year. Median prebronchodilator Forced Expiratory Volume in the first second (FEV1) increased from 3.0 L to 3.2 L (*p* < 0.001), while predicted FEV1% improved from 89.2% to 95.0% (*p* < 0.001). ACT scores rose significantly (18.0 → 24.0, *p* < 0.001), and AQLQ scores improved from 5.8 to 4.3 (*p* < 0.001). Median FEV1 increased from 3.0 L to 3.2 L (*p* < 0.001), while predicted FEV1% improved from 89.2% to 95.0% (*p* < 0.001). ACT scores rose significantly (18.0 → 24.0, *p* < 0.001), and AQLQ scores improved from 5.8 to 4.3 (*p* < 0.001).

Atopic dermatitis (AD) diagnosis was made in 19 patients according to the revised Hanifin and Rajka criteria [23]. Nineteen patients fulfilled diagnostic criteria for AD. Dupilumab produced significant clinical improvement, with median EASI decreasing from 9.3 to 0.0 (*p* = 0.015). Pruritus NRS scores dropped from 3.0 to 0.0 (*p* < 0.001), and sleep disturbance scores from 1.0 to 0.0 (*p* = 0.005). Quality of life improved substantially, with DLQI scores decreasing from 6.0 to 1.0 (*p* < 0.001).

**Figure 2:**
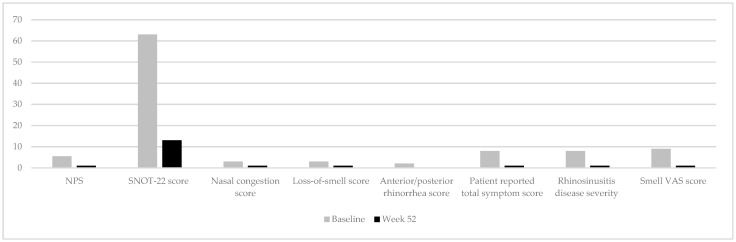
Comparison of the median values of selected outcomes measured at baseline and at week 52.

Overall, dupilumab demonstrated consistent efficacy across sinonasal disease and type 2 comorbidities (Figure 2), with clinically meaningful improvements in quality of life indices across all evaluated domains (Table 4).

### 3.2. Safety

Dupilumab was generally well-tolerated throughout the 52-week follow-up. The most commonly reported adverse events were mild injection-site reactions and transient conjunctivitis. Importantly, no serious adverse events were observed during the study period. Laboratory parameters remained stable over time, and no clinically relevant changes in peripheral eosinophil counts requiring treatment discontinuation were recorded (Table 5).

## 4. Discussion

To our knowledge, this is one of the first multicenter, prospective, real-life studies to specifically assess long-term (52-week) outcomes of dupilumab in severe CRSwNP with particular focus on coexisting multiple type 2 inflammatory comorbidities (perennial allergic rhinitis (PAR), asthma, atopic dermatitis (AD). Our findings demonstrate that dupilumab provides robust and clinically meaningful benefits across several disease domains, resulting in significant reductions in sinonasal symptom burden, nasal polyp size and markers of type 2 inflammation, while improving allergic rhinitis, asthma control, skin disease severity, and overall quality of life.

The therapeutic landscape of CRSwNP has historically relied on intranasal corticosteroids and short-term systemic corticosteroids, followed by endoscopic sinus surgery in refractory cases. However, a substantial subset of patients remains uncontrolled despite these approaches, experiencing persistent symptoms, frequent recurrences, impaired quality of life, and considerable healthcare costs [2,24,25]. This population, often defined as having “severe uncontrolled CRSwNP,” represents a significant unmet clinical need. The advent of biologic therapies targeting type 2 inflammation has revolutionized treatment paradigms, shifting the focus from symptomatic relief to disease modification.

Our multivariable regression analysis provides further insights into the determinants of therapeutic response. In the backward stepwise model, changes in patient-reported total symptom burden (ΔPOEMS) and endoscopic nasal polyp size (ΔTENPS) were the most powerful independent predictors of improvement in sinonasal-specific quality of life (ΔSNOT-22). This finding emphasizes the dual contribution of objective endoscopic outcomes and subjective symptom perception to overall treatment benefit. Moreover, the observed inverse relationship between changes in VAS scores and ΔSNOT-22 indicates that reductions in global disease perception significantly contribute to patient-reported outcomes, even beyond measurable structural changes. Although changes in peripheral eosinophil counts were statistically significant, their relatively low explanatory power suggests that systemic eosinophilia may not fully capture the local inflammatory processes that drive clinical response. These observations highlight the need for more refined, tissue-specific biomarkers—such as local cytokine signatures or transcriptomic endotypes—to better predict treatment outcomes and guide personalized biologic therapy.

The pathophysiology of CRSwNP is dominated by a type 2 inflammatory response characterized by Th2 cell activation, eosinophilic infiltration, local IgE production, and epithelial barrier dysfunction. Central to these processes are IL-4 and IL-13, key cytokines that drive B-cell class-switching to IgE, promote eosinophil recruitment and activation, and contribute to mucus hypersecretion and epithelial remodeling [7,26]. By targeting the shared IL-4Rα subunit, dupilumab simultaneously inhibits IL-4 and IL-13 signaling, thereby exerting broad immunomodulatory effects that extend beyond the upper airways and address the shared pathogenic mechanisms of multiple type 2 diseases.

In this real-world cohort, dupilumab induced marked and sustained improvements in all sinonasal parameters, including nasal polyp size, symptom burden, and sinonasal-specific quality of life. Importantly, these benefits were accompanied by significant and clinically meaningful improvements in asthma control, lung function, rhinitis severity, and atopic dermatitis activity among patients with the respective comorbidities. The consistency of these responses across heterogeneous clinical phenotypes reinforces the concept that dupilumab acts as a disease-modifying therapy targeting the common upstream drivers of type 2 inflammation.

Our real-world results align closely and extend previous findings of pivotal randomized controlled trials. In the LIBERTY NP SINUS-24 and SINUS-52 studies, dupilumab significantly improved nasal polyp score (NPS), sinonasal quality of life (SNOT-22), and olfactory function, as well as secondary outcomes such as sinus opacification and nasal congestion [27]. Similarly, the DUPIREAL observational study confirmed sustained efficacy in routine clinical practice beyond 12 months [26]. Our data build on these results by demonstrating that dupilumab retains its effectiveness in a broader, more heterogeneous patient population with multiple concomitant type 2 comorbidities—including conditions that are often underrepresented in clinical trials. These results further confirm the broad impact of dupilumab on T2 comorbidities evaluated in our previous study suggesting the suitability of the drug in patients suffering from CRSwNP and associated comorbidities [8].

Importantly, we observed significant improvements across all comorbidity subgroups. Asthma Control Test (ACT) scores and lung function (FEV_1_) increased significantly, in line with previous evidence demonstrating dupilumab’s efficacy in severe type 2 asthma [28,29]. Likewise, patients with AD experienced marked reductions in disease activity (EASI) and pruritus (NRS), with significant improvements in quality of life (DLQI), consistent with prior clinical data [30]. These results underscore the concept of shared immunopathological pathways among type 2 diseases and support the “treatable traits” approach, whereby a single biologic can address multiple disease manifestations simultaneously.

Beyond clinical efficacy, the pharmacoeconomic implications of dupilumab are noteworthy. The ability to control multiple type 2 comorbidities with a single biologic agent may reduce the need for polypharmacy, repeated corticosteroid courses, surgical interventions, and healthcare resource utilization [24]. This integrated therapeutic approach has the potential to significantly improve patient outcomes while reducing overall healthcare costs, supporting the inclusion of dupilumab as a cornerstone of personalized care for type 2 inflammatory diseases.

## 5. Conclusions

In conclusion, this prospective multicenter real-world study demonstrates that patients treated with dupilumab for CRSwNP also receive a broader and impactful collateral clinical benefit on their T2-associated comorbidities such as allergic rhinitis, bronchial asthma and atopic dermatitis. By targeting shared immunopathogenic pathways, dupilumab provides significant and clinically meaningful improvements across multiple disease domains, supporting its role as a cornerstone of precision medicine in the management of type 2 inflammatory multimorbidity.

## Figures and Tables

**Table 1:** Characteristics of patients included in the study (n = 122).

Variable	Value *	Mean (std.dev)
Age	50 ± 17	51.3 (12.5)
Sex (male)	58 (51.0)	
BMI	25 ± 5.0	25.3 (3.8)
Polyp duration (month)	84 ± 132	125 (113.2)
Polyp surgery		
=0	18 (15.9)	
≥1	95 (84.1)	
≥3	20 (17.7)	
Time since most recent polyp surgery (month)	42 ± 38	42.2 (49.5)
Allergic rhinoconjunctivitis (si) %	64 (52.4)	
Non-allergic rhinoconjunctivitis (si) %	12 (9.83)	
Bronchial asthma (si) %	66 (54.1)	
Atopic dermatitis (si) %	19 (15.6)	
Food allergy (si) %	5 (4.1)	
Chronic spontaneous urticaria (si) %	0 (0.0)	
Inhaled Steroids	24 ± 36	39.1 (46.1)

* data are median ± IQR or n (%).

**Table 2:** Change in outcome measures between baseline and 52 weeks for 74 dupilumab-treated patients with CRSwNP. Data are median ± IQR or n (%); n.s.: not significant.

Outcome	Baseline	Week 52	*p*-Value
NPS (scale 0–8)			
Mean (std dev)	5.2 (1.7)	1.0 (1.3)	
Median ± IQR	5.5 ± 2.25	1 ± 2	<0.001
SNOT-22 score (scale 1–110)			
Mean (std dev)	64.2 (22.0)	14.4 (9.9)	
Median ± IQR	63 ± 34	13 ± 13	<0.001
Nasal congestion score (scale 0–3)			
Mean (std dev)	2.6 (0.6)	0.54 (0.5)	
Median ± IQR	3 ± 1	1 ± 1	<0.001
Missing, n (%)	0 (0)	0 (0)	
Loss of smell score (scale 0–3)			
Mean (std dev)	2.6 (0.6)	0.64 (0.8)	
Median ± IQR	3 ± 1	1 ± 1	<0.001
Missing, n (%)	0 (0)	0 (0)	
*Anterior/posterior rhinorrhea score (scale 0–3)*			
Mean (std dev)	1.9 (0.9)	0.48 (0.7)	
Median ± IQR	2 ± 2	0 ± 1	<0.001
Missing, n (%)	0 (0)	0 (0)	
Patient-reported total symptom score (scale 0–9)			
Mean (std dev)	7.8 (1.3)	1.142 (1.3)	
Median ± IQR	8 ± 2	1 ± 2	<0.001
Missing, n (%)	0 (0)	0 (0)	
Rhinosinusitis disease severity (VAS 0–10 cm)			
Mean (std dev)	8.1 (1.4)	1.7 (1.9)	
Median ± IQR	8 ± 2	1 ± 3	<0.001
Missing, n (%)	0 (0)	0 (0)	
Smell (VAS 0–10 cm)			
Mean (std dev)	8.5 (1.8)	1.9 (2.4)	
Median ± IQR	9 ± 2	1 ± 3	<0.001
Missing, n (%)	0 (0)	0 (0)	
Total IgE (KUA/L)			
Mean (std dev)	557.5 (1490.0)	321.8 (480.3)	
Median ± IQR	246 ± 342.25	187 ± 329.6	0.003
Missing, n (%)	7 (6)	24 (21)	
Eosinofili (cells/mm^3^)			
Mean (std dev)	396.2 (298.1)	407.0 (337.0)	
Median ± IQR	340 ± 298.5	302 ± 269.5	0.951 n.s.
Missing, n (%)	5 (4)	*10 (8)*	

**Table 3:** Beta estimates of the regression model.

	Unstandardized Coefficients		Standardized Coefficients	t	*p*-Value
Regressors	B	Std. Error	Beta		
Constant	−2.355	8.296		−0.284	0.777
DiffTENPS	3.807	1.396	0.267	2.727	0.008
DiffVAS	−2.676	1.016	−0.273	−2.635	0.010
DiffEosin	−0.026	0.008	−0.319	−3.422	0.001
Diff_Tot_Symt	7.123	1.277	0.615	5.578	0.000
Dependent Variable: DiffSNOT					

**Table 4:** Summary of efficacy outcomes in the subgroup of patients with comorbidities at week 52 (n = 122).

Outcome	Baseline	Week 52	*p*-Value
***Perennial allergic rhinitis n = 76***			
RCSS score			
Median ± IQR	22.5 ± 11.0	12.0 ± 6.8	<0.001
Missing, n (%)	37 (32.7)		
RQLQ score			
Median ± IQR	3.0 ± 1.5	0.9 ± 1.4	<0.001
Missing, n (%)	37 (32.7)		
***Bronchial asthma n = 58***			
FEV1 (L) before bronchodilation (liters)			
Median ± IQR	3.0 ± 1.1	3.2 ± 1.1	<0.001
Missing, n (%)	55 (48.7)		
FEV1% of predicted.			
Median ± IQR	89.2 ± 17.9	95.0 ± 14.0	<0.001
Missing, n (%)	55 (48.7)		
ACT score	(n = 62)		
Median ± IQR	18.0 ± 6.3	24.0 ± 2.0	<0.001
Missing, n (%)	51 (45.1)		
AQLQ (S) score	n = 62		
Median ± IQR	5.8 ± 1.2	4.3 ± 2.3	<0.001
Missing, n (%)	51 (45.1)		
***Atopic dermatitis n = 19***			
EASI score			
Median ± IQR	9.3 ± 21.5	0.0 ± 2.5	0.015
Missing, n (%)	94 (83.2)		
NRS pruritus			
Median ± IQR	3.0 ± 7.0	0.0 ± 1.0	0.001
Missing, n (%)	94 (83.2)		
NRS for sleep			
Median ± IQR	1.0 ± 8.0	0.0 ± 0.0	0.005
Missing, n (%)	94 (83.2)		
DLQI score			
Median ± IQR	6.0 ± 20.0	1.0 ± 2.0	0.000
Missing, n (%)	94 (83.2)		

**Table 5:** Adverse events observed during 52 weeks of dupilumab treatment in patients with chronic rhinosinusitis with nasal polyps (n = 122).

Adverse Event	Number of Patients, n (%)	Severity	Outcome
Conjunctivitis	1 (0.8%)	Mild	Resolved with topical treatment; no treatment discontinuation
Injection-site reaction (erythema)	2 (1.6%)	Mild	Transient; no treatment discontinuation
Transient asymptomatic hypereosinophilia	1 (0.8%)	Mild	Spontaneous resolution; no clinical consequences
Serious adverse events	0 (0%)	—	—

## Data Availability

The original contributions presented in this study are included in the article. Further inquiries can be directed to the corresponding author.

## References

[1] Galletti C., Ciodaro F., Barbieri M.A., Gambino F., Ferrisi M.G., Portelli D., Catalano N., Spina E., Freni F., Galletti B. (2024). Effectiveness and safety profile of mepolizumab in chronic rhinosinusitis with nasal polyps: Real life data in a tertiary care. Am. J. Otolaryngol.—Head Neck Med. Surg..

[2] Fokkens W.J., Lund V.J., Hopkins C., Hellings P.W., Kern R., Reitsma S., Toppila-Salmi S., Bernal-Sprekelsen M., Mullol J., Alobid I. (2020). European Position Paper on Rhinosinusitis and Nasal Polyps 2020. Rhinology.

[3] Bachert C., Hellings P.W., Mullol J., Naclerio R.M., Chao J., Amin N., Grabher A., Swanson B.N., Hamilton J.D., Guillonneau S. (2019). Dupilumab improves patient-reported outcomes in patients with chronic rhinosinusitis with nasal polyps and comorbid asthma. J. Allergy Clin. Immunol. Pract..

[4] Marcus S., Roland L.T., DelGaudio J.M., Wise S.K. (2018). The relationship between allergy and chronic rhinosinusitis. Laryngoscope Investig. Otolaryngol..

[5] Canonica G.W., Bourdin A., Peters A.T., Desrosiers M., Bachert C., Weidinger S., Simpson E.L., Daizadeh N., Chen Z., Kamat S. (2022). Dupilumab Demonstrates Rapid Onset of Response Across Three Type 2 Inflammatory Diseases. J. Allergy Clin. Immunol. Pract..

[6] Papapostolou N., Xepapadaki P., Katoulis A., Makris M. (2022). Comorbidities of Chronic Urticaria: A glimpse into a complex relationship. Front. Allergy.

[7] Harb H., Chatila T.A. (2020). Mechanisms of Dupilumab. Clin. Exp. Allergy.

[8] Nettis E., Brussino L., Patella V., Bonzano L., Detoraki A., Di Leo E., Sirufo M.M., Caruso C., Lodi Rizzini F., Conte M. (2022). Effectiveness and safety of dupilumab in patients with chronic rhinosinusitis with nasal polyps and associated comorbidities: A multicentric prospective study in real life. Clin. Mol. Allergy.

[9] Gazzetta n. 262 del 8 Novembre 2024—AGENZIA ITALIANA DEL FARMACO. http://www.gazzettaufficiale.biz/atti/2024/20240262/24A05865.htm.

[10] Fokkens W.J., Viskens A.S., Backer V., Conti D., De Corso E., Gevaert P., Scadding G.K., Wagemann M., Bernal-Sprekelsen M., Chaker A. (2023). EPOS/EUFOREA update on indication and evaluation of Biologics in Chronic Rhinosinusitis with Nasal Polyps 2023. Rhinology.

[11] Mauthe T., Ryser F.S., Brühlmann C., Yalamanoglu A., Meerwein C., Steiner U.C., Soyka M.B. (2025). Correlation of sino-nasal outcome test and nasal polyp score in dupilumab-treated chronic rhinosinusitis with nasal polyps. Eur. Arch. Otorhinolaryngol..

[12] Hopkins C., Gillett S., Slack R., Lund V.J., Browne J.P. (2009). Psychometric validity of the 22-item Sinonasal Outcome Test. Clin. Otolaryngol..

[13] Kim D.H., Stybayeva G., Hwang S.H. (2024). Comparative Effectiveness of Dupilumab Versus Sinus Surgery for Chronic Rhinosinusitis With Polyps: Systematic Review and a Meta-Analysis. Am. J. Rhinol. Allergy.

[14] Mullol J., Bachert C., Amin N., Desrosiers M., Hellings P.W., Han J.K., Jankowski R., Vodicka J., Gevaert P., Daizadeh N. (2022). Olfactory Outcomes With Dupilumab in Chronic Rhinosinusitis With Nasal Polyps. J. Allergy Clin. Immunol. Pract..

[15] Crimi C., Campisi R., Noto A., Genco S., Cacopardo G., Nolasco S., Crimi N. (2020). Comparability of asthma control test scores between self and physician-administered test. Respir. Med..

[16] Bateman E.D., Esser D., Chirila C., Fernandez M., Fowler A., Moroni-Zentgraf P., FitzGerald J.M. (2015). Magnitude of effect of asthma treatments on Asthma Quality of Life Questionnaire and Asthma Control Questionnaire scores: Systematic review and network meta-analysis. J. Allergy Clin. Immunol..

[17] Hanifin J.M., Thurston M., Omoto M., Cherill R., Tofte S.J., Graeber M. (2001). The eczema area and severity index (EASI): Assessment of reliability in atopic dermatitis. EASI Evaluator Group. Exp. Dermatol..

[18] Lee H.J., Yuri W.O.O., Lee Y.B., Lee J.H., Kim J.E., Lee J.H., Cho S.H. (2025). Evaluating the Real-World Effectiveness of Systemic Treatments in Atopic Dermatitis Using the Atopic Dermatitis Control Tool (ADCT): A Multi-Centre, Prospective Study. Acta Derm. Venereol..

[19] Yosipovitch G., Reaney M., Mastey V., Eckert L., Abbé A., Nelson L., Clark M., Williams N., Chen Z., Ardeleanu M. (2019). Peak Pruritus Numerical Rating Scale: Psychometric validation and responder definition for assessing itch in moderate-to-severe atopic dermatitis. Br. J. Dermatol..

[20] Salameh P., Gutsche A., Aulenbacher F., Buttgereit T., Weller K., Siebenhaar F., Maurer M. (2024). Urticaria Control Test real-world performance: A post-hoc analysis. Allergy.

[21] Downloading IBM SPSS Statistics 25. https://www.ibm.com/support/pages/downloading-ibm-spss-statistics-25.

[22] 2024 GINA Main Report—Global Initiative for Asthma—GINA. https://ginasthma.org/2024-report/#.

[23] Hanifin J.M., Cooper K.D., Ho V.C., Kang S., Krafchik B.R., Margolis D.J., Schachner L.A., Sidbury R., Whitmore S.E., Sieck C.K. (2004). Guidelines of care for atopic dermatitis. J. Am. Acad. Dermatol..

[24] Chen S., Zhou A., Emmanuel B., Thomas K., Guiang H. (2020). Systematic literature review of the epidemiology and clinical burden of chronic rhinosinusitis with nasal polyposis. Curr. Med. Res. Opin..

[25] Hellings P.W., Akdis C.A., Bachert C., Bousquet J., Pugin B., Adriaensen G., Advani R., Agache I., Anjo C., Anmolsingh R. (2017). EUFOREA Rhinology Research Forum 2016: Report of the brainstorming sessions on needs and priorities in rhinitis and rhinosinusitis. Rhinology.

[26] De Corso E., Pasquini E., Trimarchi M., La Mantia I., Pagella F., Ottaviano G., Garzaro M., Pipolo C., Torretta S., Seccia V. (2023). Dupilumab in the treatment of severe uncontrolled chronic rhinosinusitis with nasal polyps (CRSwNP): A multicentric observational Phase IV real-life study (DUPIREAL). Allergy.

[27] Bachert C., Han J.K., Desrosiers M., Hellings P.W., Amin N., Lee S.E., Mullol J., Greos L.S., Bosso J.V., Laidlaw T.M. (2019). Efficacy and safety of dupilumab in patients with severe chronic rhinosinusitis with nasal polyps (LIBERTY NP SINUS-24 and LIBERTY NP SINUS-52): Results from two multicentre, randomised, double-blind, placebo-controlled, parallel-group phase 3 trials. Lancet.

[28] Pelaia C., Benfante A., Busceti M.T., Caiaffa M.F., Campisi R., Carpagnano G.E., Crimi N., D’Amato M., Foschino Barbaro M.P., Maglio A. (2023). Real-life effects of dupilumab in patients with severe type 2 asthma, according to atopic trait and presence of chronic rhinosinusitis with nasal polyps. Front. Immunol..

[29] Minagawa S., Araya J., Watanabe N., Fujimoto S., Watanabe J., Hara H., Numata T., Kuwano K., Matsuwaki Y. (2022). Real-life effectiveness of dupilumab in patients with mild to moderate bronchial asthma comorbid with CRSwNP. BMC Pulm. Med..

[30] Sroka-Tomaszewska J., Bulińska B., Wilkowska A., Nowicki R.J., Trzeciak M. (2023). Dupilumab for the treatment of moderate and severe atopic dermatitis: Real-life experience. Postepy Dermatol. Alergol..

